# Thermal Ecology of a Central Anatolian Population of *Ophisops elegans*: Insights From Field Measurements and Microclimate Modeling

**DOI:** 10.1002/ece3.73518

**Published:** 2026-04-17

**Authors:** Sezer Dıblan, Arda Cem Kuyucu, Mehmet Kürşat Şahin, Selim Sualp Çağlar

**Affiliations:** ^1^ Biology Department Hacettepe University Ankara Turkey; ^2^ Biology Department Namık Kemal University Tekirdağ Turkey

**Keywords:** microclimate, *Ophisops elegans*, thermal ecology, thermoregulation

## Abstract

*Ophisops elegans*, a widespread lizard species, serves as an ideal model organism for investigating how environmental and morphological parameters influence the thermal ecology of lizards. In this study, the thermal ecology of *O. elegans* was examined from a single population from central Turkey by recording body temperatures alongside various morphological and environmental parameters. Additionally, microclimate temperatures were measured using dataloggers and modeled using NicheMapR. Thermoregulatory efficiency was calculated separately using field recorded and NicheMapR modeled temperatures. As expected, substrate and air temperatures emerged as the most significant factors influencing thermal biology, with body size further modulating thermal dynamics through interactions with substrate temperature. Thermoregulatory efficiency values remained biologically stable across the season (*E* ~ 0.78), with field recorded and modeled thermal indexes being very similar, validating NicheMapR as a robust tool for characterizing the thermal characteristics of ectotherms. These findings provide a critical baseline for forecasting the response of *O. elegans* populations to shifting climatic regimes.

## Graphical abstract

Thermal ecology and behavior of *O. elegans* were examined with related morphological and environmental parameters. Substrate and air temperatures are the main factors influencing thermal biology. Thermoregulatory efficiency values indicate that *O. elegans* is an effective thermoregulator.

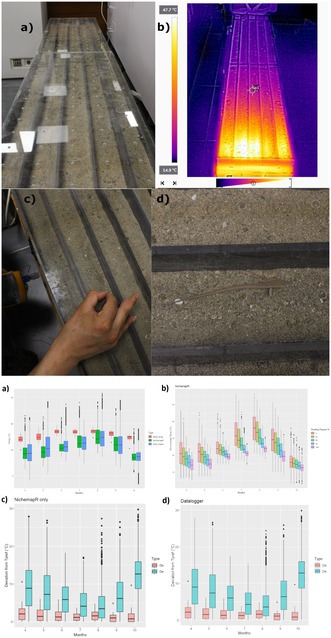

## 1 Introduction

As ectotherms, reptiles mainly depend on behavior to keep their body temperature at optimal limits in variable thermal environments for maintaining homeostasis and continuation of other biological activities and processes (Somero 2004; Angilletta 2009; Barroso et al. 2016; Li et al. 2017; Sagonas et al. 2017). Lizards implement different behavioral strategies such as shuttling, basking and orientation to regulate their body temperature by utilizing solar radiation, conduction (with the substrate), and convection (with air) (Dreisig 1984; Sagonas et al. 2017). Individuals shuttle between the open sun and shady cool refuges regularly to maximize their fitness and prevent overheating (Corbalán et al. 2013; Sinervo et al. 2010).

The interaction between morphology and environmental parameters is also important, especially body size, which is suggested to be one of the determinants of thermoregulation in reptiles (Garrick 2008; Ortega and Martín‐Vallejo 2019). One explanation assumes that this effect is more prominent in smaller‐sized ectotherms, as they have a larger surface‐to‐volume ratio and lower thermal inertia, causing them to heat and cool more rapidly compared to larger ectotherms (Rubalcaba et al. 2019; Sagonas, Meiri, et al. 2013). Thus, they rely greatly on behavioral thermoregulation compared to larger ectotherms, which have more stable body temperatures due to higher thermal inertia (Alford and Lutterschmidt 2012; Bulté and Blouin‐Demers 2010). On the other hand, in hot environments, larger ectotherms risk overheating as they generally reach higher body temperatures and are not able to cool off as quickly compared to smaller organisms (Rubalcaba et al. 2019). Yet, the effect of body size is not fully observed in reptiles, for example mass was not a significant predictor in a large scale study on squamates by Dubiner et al. (2024). Additionally the nonconforming patterns of body size in terrestrial vertebrate ectotherms also question the role of body size in heat conservation (Olalla‐Tárraga et al. 2006; Pincheira‐Donoso et al. 2008; Feldman and Meiri 2014). On the other hand, the joint of effects of size and size‐imposed microclimate should not be ignored in thermal ecology investigations of ectotherms as body size can determine body temperature both by its direct effect on the heating rate and also by its role in the microclimate experienced (Kearney et al. 2020).

Microclimate is a crucial component for thermal biology studies of ectotherms (Kemppinen et al. 2024) and most thermoregulation studies mainly depend on measurements from models and dataloggers for a uniform regular distribution of effective temperatures of microclimates in the field. In recent years there has been significant advance in methods in modeling of microclimate in ecology led by remarkable packages like NicheMapR (Kearney and Porter 2020) and Microclima (Maclean et al. 2019) and more accurate high‐resolution microclimate data became available for use in thermal ecology studies on ectotherms. Numerous thermal biology studies successfully implemented parameters derived from these microclimate models (Enriquez‐Urzelai et al. 2020; Huang et al. 2020; Rubalcaba et al. 2019; Turner and Maclean 2022).

*Ophisops elegans* is a widespread species that is commonly referred to as the snake‐eyed lizard, distributed in southeast Europe, Anatolia, and the Middle East, which is divided into several clades and exhibits remarkable diversity, with genetic lineages that exhibit distinctive variations (Agarwal and Ramakrishnan 2017; Kyriazi et al. 2008; Montgelard et al. 2020). *O. elegans* is a heliothermic diurnal species that can be found in different types of habitats in the Mediterranean and steppe‐type environments with a relatively long activity period from early spring to autumn. Additionally, the relatively small body size and the high abundance make it a convenient model organism for thermal biology studies. Here, we aimed to investigate the thermal biology of *O. elegans* using a single population from central Anatolia by setting four hypotheses. First, we suggest that the field body temperature (*T*_b_) is primarily influenced by certain environmental factors (Hypothesis 1), namely solar radiation (*Sol*), air temperature (*T*_a_), and substrate temperature (*T*_s_), which are primary determinants of field body temperature (*T*_b_). Secondly, as expected from a small ectotherm, we hypothesize that body size significantly influences thermoregulation behavior (Hypothesis 2), particularly body mass (*Mass*) and body length (*SVL*). Third, we propose that *O. elegans* , as a small heliothermic ectotherm, will exhibit significant behavioral thermoregulation efficiency (Hypothesis 3); expected to be greater than 0.7, thus making it an efficient thermoregulator rather than a thermoconformer. Additionally, we aim to investigate how seasonal differences affect the thermoregulatory efficiency. Our final aim is the validation of the reliability of the mechanistic modeling approach in characterizing the thermal niche of *O. elegans*. Thus, we assume that NicheMapR, a mechanistic microclimate modeling framework, will provide close results with field recorded microclimate temperatures, and additionally we propose that thermoregulation indexes calculated from operational temperatures (*T*_e_) provided by NicheMapR should not differ significantly from those calculated using field data loggers (Hypothesis 4). Given that the accuracy of mechanistic models often depends on the structural complexity of the habitat, validating these tools in a thermally diverse steppe environment presents a good opportunity to test if they reflect the actual thermal constraints faced by the population.

## 2 Material and Methods

### 2.1 Study Area

To better monitor *O. elegans* and field parameters regularly, we selected a single population location close to the research center. The study area is located in Hacettepe University Beytepe Campus in Ankara province of Turkey, 39°51′23″ N 32°44′26″ E, 975–1025 m elevation. The total perimeter of the study area is 1.63 km and its area is approximately 4.45 ha. Located in Central Anatolia, Ankara province has a continental climate that has cold winters with rain and snow, and hot and dry summers. The hottest months are July and August with a highest temperature value of 40.8°C, while the coldest month is January with a lowest temperature of −24.9°C (TSMS 2024). Over the years, conifer trees, especially black pine ( *Pinus nigra* ) and cedar (*Cedrus*), and various deciduous Rosaceae species such as *Prunus* have been planted with the intention of afforestation in the area. The natural steppe vegetation of Beytepe exists in the clearings and gaps of pine trees, which are very dense in some places. Although it is covered with very short and dry grass in the autumn, it has been observed that in the spring herbaceous plants grow up to half a meter, and various suitable heterogeneous micro‐habitats for lizards are formed which makes the area a convenient model for behavioral thermoregulation studies. The steppe vegetation of the area consists of chamaephyte plants such as *Astragalus microcephalus* & *A. angustifolius* , and hemicryptophyte plants such as *Verbascum ancyritanum*, *V. stachydifolium*, *V. vulcanicum* & *Anchusa azurea* . Besides, *Crocus ancyrensis*, *Pastanica sativa*, *Brassica elongata* , *Papaver rhoeas* , *Thymus zygoides*, and *Lamium album* can be given as examples of herbaceous plants in the region, *Amygdalus orientalis* and *Jasminum fruticans shrubs* are among other plants in the vegetation (Erik 1994).

### 2.2 Field Measurements

Lizard samplings were carried out at designated stations within the Beytepe Campus for 4–7 days each month between April 1 and November 1 of 2022. The main approach in these studies followed previous studies on Lacertidae (Ortega and Pérez‐Mellado 2016; Rowe et al. 2020; Sagonas et al. 2017; Şahin and Kuyucu 2021). In order to prevent the effect of increased activity on body temperature, animals were captured within 1 min after detection. Body temperature (*T*_b_) of animals was measured from the cloaca with a T‐type thermocouple connected to a Fluke 54 II thermometer. To minimize the thermal effect of the handler, the researcher handling the lizards wore insulated work gloves, and care was taken to ensure that gloves did not get too hot or cold during fieldwork. All captures were made in the same direction in the field to prevent recapture of the same individuals on the same day. After the body temperature measurements, *SVL* was measured using a digital caliper (0.01 mm precision) and body mass was recorded using a portable digital balance (0.01 g precision).

**FIGURE 1:**
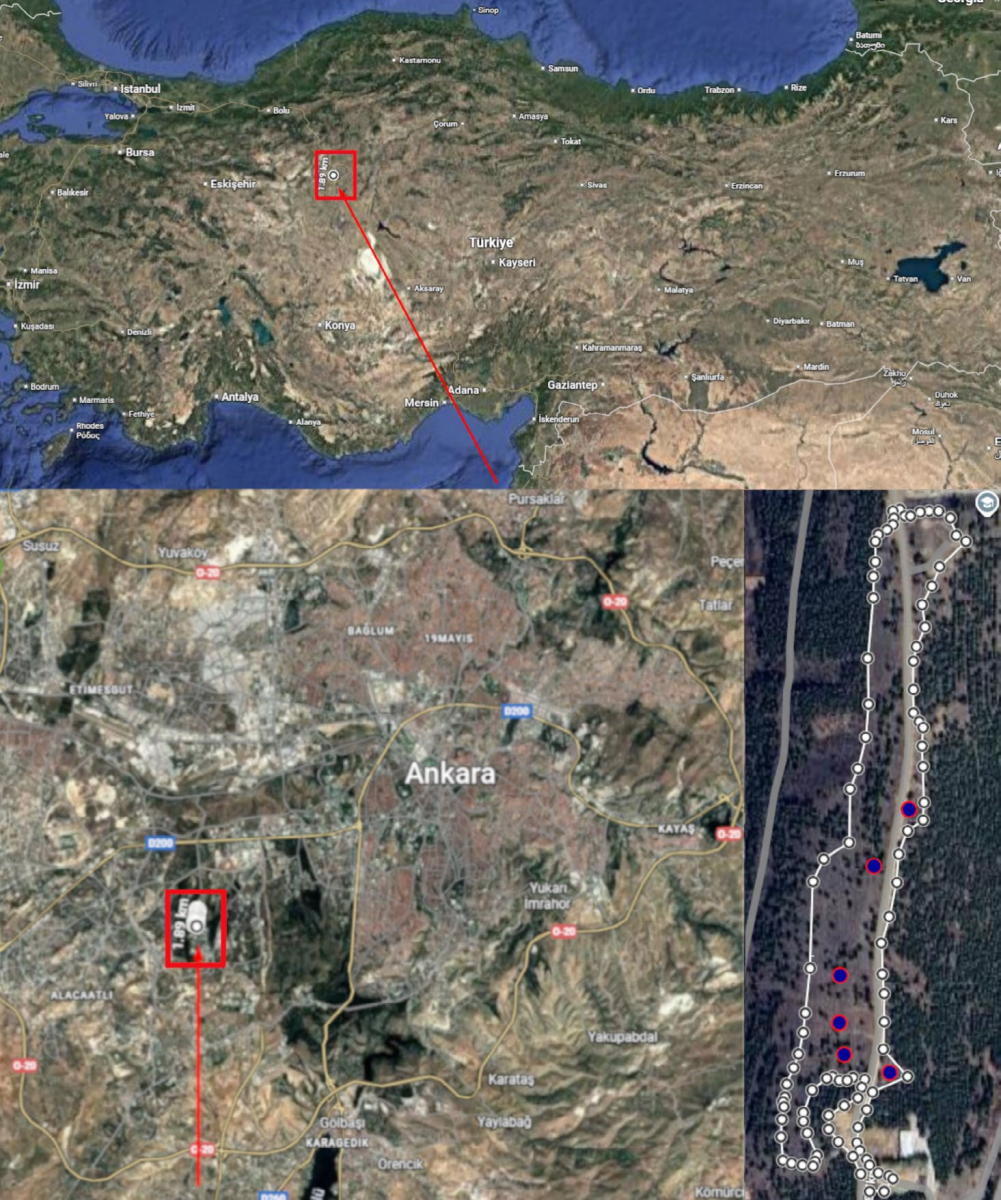
Maps showing the study region with the outlined study area and datalogger locations (blue points) in the bottom right map.

Microclimate temperatures were recorded in 10‐min intervals during the same activity period of *O. elegans* between April 1 and November 1 2022 with 6 rectangular plastic Hobo MX100 dataloggers (6.9 × 4.5 × 1.1 cm, 25.5 g) placed in the field under rock, bush bottom, tree bottom, open soil (2), and in the grass vegetation, these sites were chosen according to observations of activity and retreat places of *O. elegans* in the field. Although 6 dataloggers might seem to be a small number, the study area is not a large area and care was given to place dataloggers in regular intervals (Figure 1). Additionally, The temperature data were filtered to include only the activity hours between 06:00 AM and 18:00 PM. The total number of datalogger recordings amounted to 58,694 after the initial filtering for a total of 214 days. Using dataloggers embedded in physical models representing lizards is a widely utilized method for operative temperature in thermoregulation studies of reptiles (Ortega et al. 2017; Sagonas et al. 2017). However, *O. elegans* is a very small lizard species with an even smaller mass than the dataloggers used in the study and our dataloggers were not suitable for fitting into physical models. Considering the biophysical properties of lizards (Fei et al. 2012) dataloggers do not replicate the thermal properties of the lizards, such as body shape, mass, and heat capacity, thus to get the estimates of effective temperatures (*T*_e_) we calculated the predicted body temperatures for a passive reptile with 2.4 g body mass (field mean value from this study) with datalogger recorded temperature for microclimate using the core heat algorithm ectoR_devel included in NicheMapR package (Kearney and Porter 2020) available at https://github.com/mrke/NicheMapR/blob/master/R/ectoR_devel.R. As the main aim of *T*_e_ to provide a standardized simplified assumption of operative environmental temperatures we used the mean body mass from the field as a single value. ectoR_devel is a modular implementation of the heat budget of the Niche Mapper ectotherm model that computes body temperature (Kearney and Leigh 2024). During field captures, after determining the substrate type, the substrate temperature (*T*_s_) was taken from the point where the individual was caught with the same contact‐sensitive thermometer, and solar radiation (watts/m^2^) was measured with a pyranometer (Apogee mp100) from the point where the individual was detected. In addition, simultaneously with determining the substrate temperature values in the environment, wind speed (m/s) and air temperature (*T*_a_) (0.1°C precision) were measured with an UNI‐T anemometer thermometer. The determined substrate types active lizards were caught are grouped under three categories as soil, grass, and rock.

### 2.3 Modeling the Microclimate Temperatures

In addition to recording the microclimate temperatures using dataloggers, microclimate temperatures of the study location during the sampling and activity period were modeled with the NicheMapR package (Kearney and Porter 2020). Required climatic parameters were downloaded from ERA5 (Hersbach et al. 2023) with the mcera5 function which is also included in this package. Using the micro_era5 function, we modeled the hourly microhabitat temperatures for 0%, 25%, 50%, 75%, and 100% shade conditions between April and November 2022 during the activity hours of 06:00 to 18:00 for 2.5 cm height above ground (the presumed height of *O. elegans* ).

### 2.4 Thermal Preference Assays

The lizards captured for temperature preference measurements were housed in a terrarium (20 × 36 × 17 cm) overnight to acclimate to in vitro conditions; they were fed with arthropods such as ants and mosquitoes, and their water ad libitum. An under‐heater mat was provided under a sandy substrate, and a halogen lamp was used during daylight hours to maintain a temperature of ~25°C. A small box was placed horizontally in the terrarium to create shade and a place where individuals could hide other than substrates.

**FIGURE 2:**
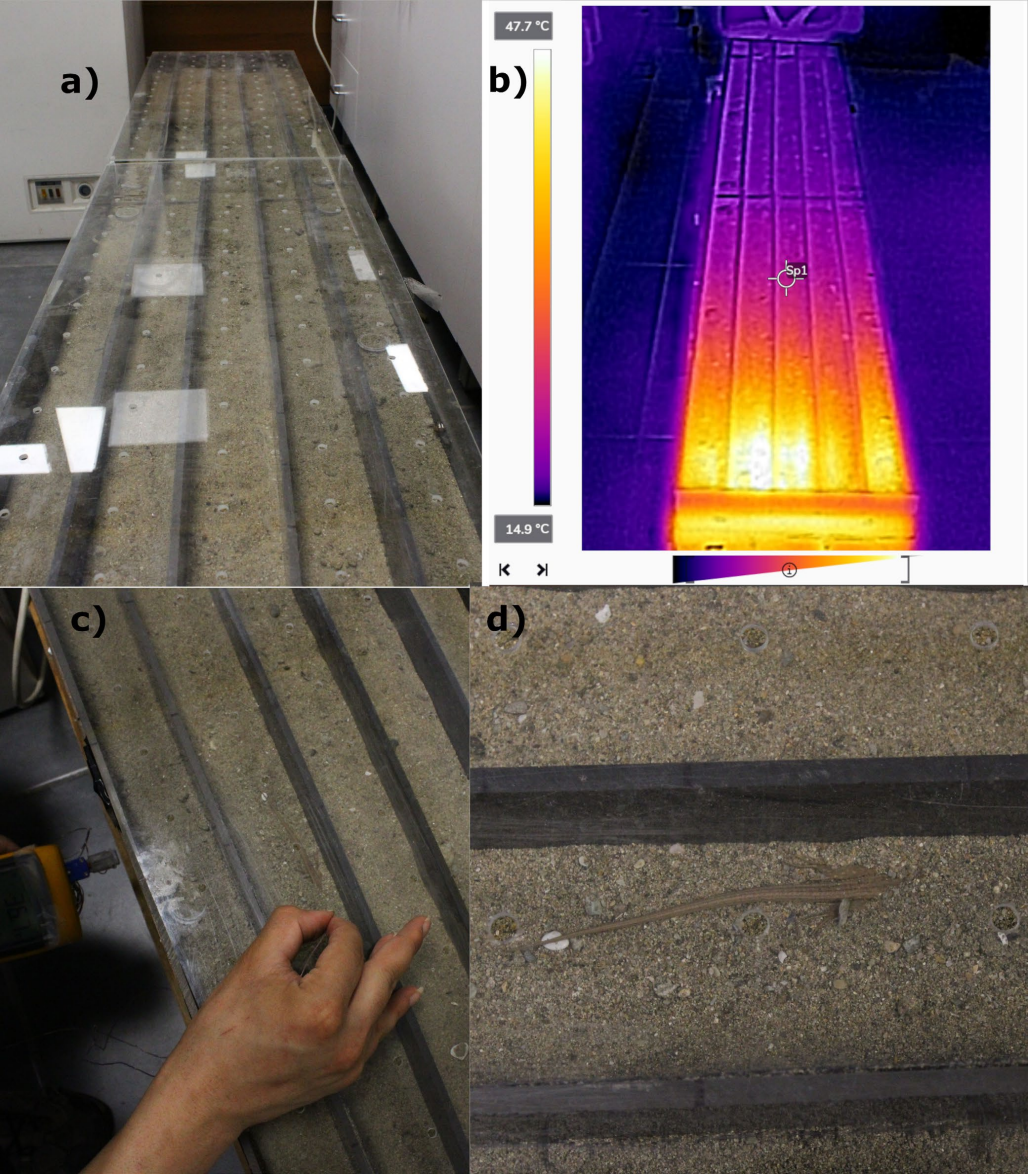
(a) Thermal gradient setup from above. (b) Thermal image of the gradient taken with a thermal camera (Flir C3) (c) Insertion of thermocouples during measurements. (d) Individual lizard in the thermal gradient, half burrowed in sand.

A thermal gradient setup was used for thermal preference measurements according to Kuyucu and Çağlar (2016). The thermal gradient consists of a copper plate of 150 × 30 cm divided into five lanes with 5 cm high panels, during experiments the gradient is topped by a transparent plexiglass cover that has regular holes for measurements. One end was cooled with ice packs while the other end was heated with an adjustable thermostat. Each lane was provided with a 5 mm thick sand substrate and the temperature ranged from ~12°C to 45°C (Figure 2). After the system reached equilibrium and then after waiting for a 30 min adjustment period, a total of 8 measurements were done at 15‐min intervals, *T*_pref_ for each individual was taken as the mean of these 8 measurements. In order to prevent the individual from leaving the position where the measurement was taken, the individual was approached in a way that disturbed it as little as possible and measurements were taken from its dorsal skin and from the location of the lizard with the same contact thermocouple used in field measurements by inserting from the regular openings (sensitivity of 0.1°C). Measuring body temperature from the dorsal area using infrared (IR) thermography is noninvasive and causes minimal disturbance to the reptiles, making it a practical method for field studies (Barroso et al. 2016) additionally the gradient is closed from the top and in addition constant recapturing during a relatively short might disrupt thermoregulatory behavior (Camacho and Travis 2017). Generally, cloacal temperatures and dorsal temperatures may show differences near 2°C for species like *Podarcis virescens* (Barroso et al. 2016) under an infrared heat source on the other this difference was much smaller in study on iguanas and chameleons which did not involve an infrared heat source (Cremer et al. 2019). When the measurements were completed, individuals were released to the exact location where they were taken. Due to the limitations of the housing conditions of the research laboratory we had to limit thermal preference measurements to a total of 23 individuals (*n* = 13 males, 7 juveniles and 3 females) collected in September. Fieldwork and collection of lizards for laboratory examinations in Beytepe, Türkiye was done under the permit no. 52338575 from the Animal Research Ethics Committee of Hacettepe University.

### 2.5 Thermoregulation Efficiency

In order to assess the effectiveness of thermoregulation, the method first proposed by (Hertz et al. 1993) was followed. Our aim was first to calculate the overall thermoregulation efficiency for this population from field (datalogger) derived and modeled (NicheMapR) parameters and secondly compare efficiencies between field and modeled. Firstly, the 50% interquartile range of *T*_pref_ was calculated and the deviation of *T*_b_ from *T*_pref_ (*d*_b_) was set as 0 if it falls within this range and if the difference falls outside these margins, the absolute difference was used. Deviation of environmental temperatures from *T*_b_ (*d*_e_) followed the same procedure and was calculated separately for *T*_e_ values gathered from dataloggers in the microclimate and *T*_e_ values gathered from NichemapR models. Then the accuracy of thermoregulation was calculated as follows:

$$E=1-\frac{\underline{\bar{d_{b}}}}{d_{e}}$$

Since the effectiveness of thermoregulation (*E*) is a single value, to provide random distributions for *E* values, a two‐step bootstrap procedure was followed; (1) 1000 temperature points from the recorded and modeled environmental temperature pools were sampled from datalogger data and NichemapR models and thermoregulation effectiveness (*E*) was calculated (2) this sampling and calculation of *E* was repeated randomly 1000 times to build distributions with 95% confidence for comparisons.

### 2.6 Statistical Analysis

All statistical analyses were carried out with R software (R Core Team 2022).

#### 2.6.1 Factors Affecting Body Temperature in the Field

To investigate the effect of several environmental and morphological parameters on the body temperature (*T*_b_) of *O. elegans* , a generalized additive model (GAM) approach was followed using the mgcv package for R (Wood and Wood 2015). A preliminary model was built with all morphological and environmental parameters and important possible interrelations between the parameters. In the model, *T*_b_ was the response variable while gender and substrate type were taken as categorical predictors. Snout vent length (*SVL*), body mass (*Mass*), air temperature (*T*_a_), wind speed (*Wind*), substrate temperature (*T*_s_), and solar radiation (*Sol*) were added to the model as continuous predictors with Gaussian smooths, while Julian day of the year and time of day in minutes were included in the model with cyclic cubic splines. Included interactions between variables were (*SVL*, *T*_a_), (*Sol*, *SVL*), (*Mass*, *T*_s_), (*Mass*, *T*_a_), (*Mass*, *SVL*), and (*T*_a_, *T*_s_). After building the initial model with all the above parameters and interactions, an Akaike Information Criteria (Aho et al. 2014; Burnham and Anderson 2004) procedure was followed with backward stepwise eliminating the first nonsignificant parameter from the model; if the model AIC was improved after elimination, this simpler model was chosen and the same procedure was continued with the next candidate parameter until no further improvement to the model was possible. In case of no improvement, the chosen parameter was left in the model and the procedure was continued with the next candidate parameter. All models were checked with the gam.check() function included in the mgcv package.

#### 2.6.2 Comparison of Field Derived and NichemapR Modeled Microclimate Parameters

Datalogger derived and NichemapR modeled temperatures were also compared between each other for each month by equivalence testing with a TOST procedure, using two (upper bound and lower bound) one‐sided *t*‐tests were also used to test for equivalence using 1°C margin as threshold using the toster package for R (Lakens and Caldwell 2018). It should be taken into consideration that in an equivalence test with a TOST procedure the null hypothesis is two samples differ between each other greater than the set margin, two tests are employed for upper and lower bound and if the *p* value is significant for both tests it means that two samples do not differ between each other greater than the set margin (1°C in our tests) if one of these tests fails to reject the null hypothesis (*p* > 0.05) the outcome is that the samples differ from each other (Richter and Richter 2002).

#### 2.6.3 Thermal Preference and Thermoregulation

To compare *T*_pref_ between different sexes (males, females, and juveniles) a Kruskal–Wallis test was used due to the nonnormal distribution of the data and the relatively small sample size. To compare thermoregulation indexes built with datalogger derived and NicheMapR modeled temperature parameters a *t*‐test was utilized to analyze the difference between the two bootstrap sampled distributions. A nested ANOVA was carried out to investigate the seasonal effect on thermoregulation with the first grouping factor being data source (Datalogger vs. NicheMapR) and the second factor being months. Additionally, we carried out another equivalence test procedure to test the difference between datalogger derived and NicheMapR modeled thermoregulation efficiency for all months; for this equivalence test procedure we set a difference boundary margin of 5%.

## 3 Results

### 3.1 Field Measurements

**FIGURE 3:**
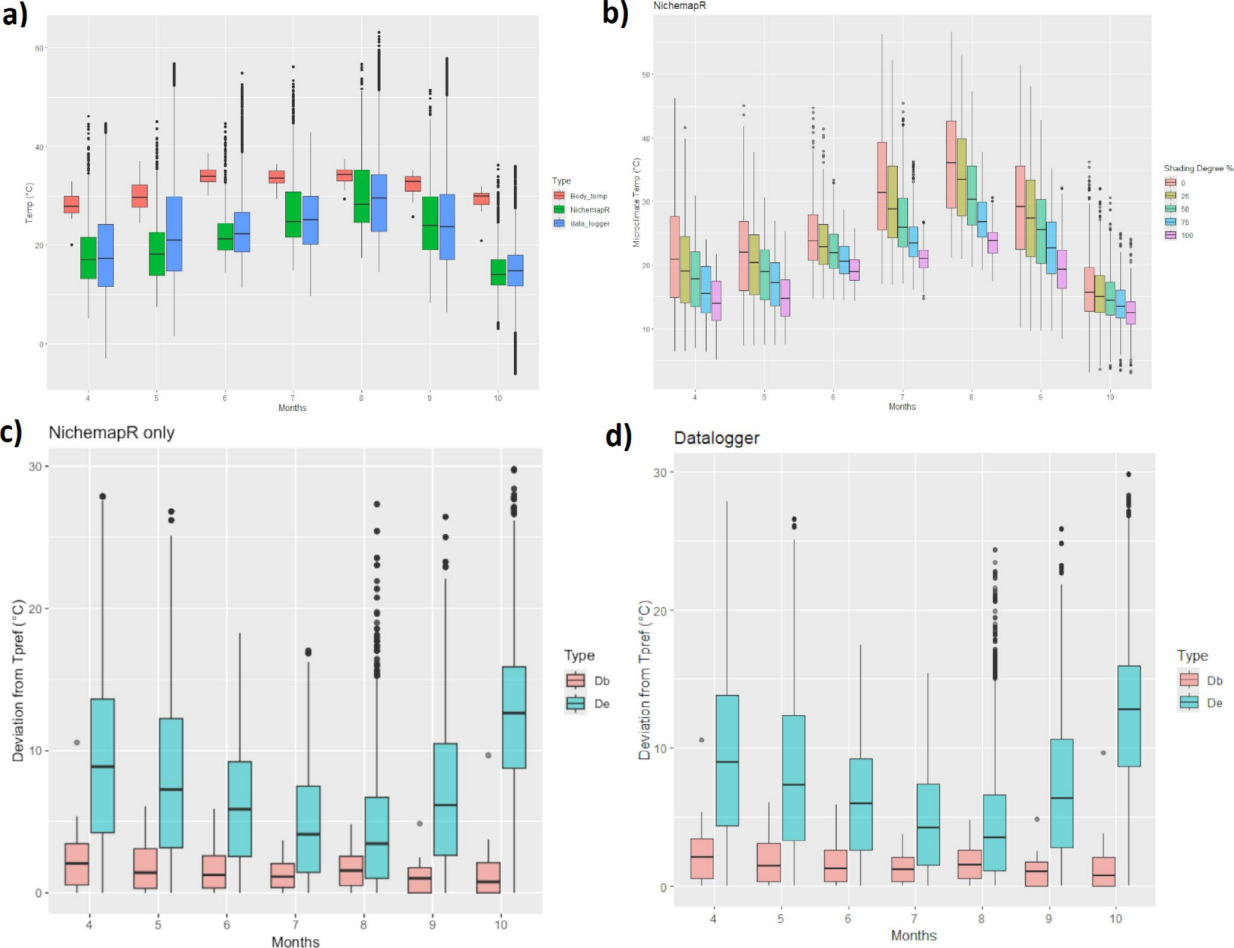
(a) Body temperatures of *O. elegans* with datalogger recorded and NicheMapR modeled microclimate temperatures in activity months. (b) NicheMapR modeled temperatures for different shade conditions. (c) Deviation of body and modeled environmental temperatures from *T*_pref_ (d) Deviation of body and datalogger recorded environmental temperatures from *T*_pref_.

The results of field body temperature measurements are shown in Table 1 and the results of morphological measurements are shown in Table 2. As expected, environmental and body temperatures show a significant increase in summer and a decrease in autumn (Figure 3a). Microclimate temperatures taken from dataloggers and the microclimate temperatures taken from NicheMapR (mean of 0–100% shade conditions) were relatively close (Figure 3a) with a mean overall difference of 0.41°C (TOST, *p* < 0.05 for both tests for a margin of 1°C). On the other hand, a significant difference was observed when the differences were compared by months. May (Month 5) showed the greatest difference of 4.17°C followed by July (1.73°C difference) and June (1.32°C difference); equivalence tests also failed for these 3 months (TOST, *p* > 0.05), while the difference was smaller than 1°C for other months.

**TABLE 1:** Body temperature measurements (*T*_b_) in the field and thermal preference measurements (*T*_pref_).

	*T*_b_ (°C) ± St. Dev.	*T*_pref_ (°C) ± St. Dev.
Female	33.25 ± 1.96 (range 29.2–36.5, *n* = 16)	33.91 ± 1.76 (range 32.94–35.84, *n* = 3)
Male	32.59 ± 3.32 (range 20.9–38.6, *n* = 76)	31.72 ± 2.61 (range 26.48–35.25, *n* = 13)
Juvenile	30.9 ± 2.76 (range 24.8–37.5, *n* = 48)	32.03 ± 1.73 (range 29.71–34.54, *n* = 7)
All	32.17 ± 3.13 (range 20.9–38.6, *n* = 140)	32.10 ± 3.11 (range 26.48–35.84, *n* = 23)

**TABLE 2:** Morphological measurements in the field.

Measurement	Max	Min	Mean ± St. Dev.
All SVL (mm)	58.5	32.7	46.2 ± 5.97
Female SVL (mm)	52.29	45.03	49.38 ± 2.25
Male SVL (mm)	58.5	33.39	49.77 ± 3.62
Juvenile SVL (mm)	44.65	32.73	39.10 ± 3.09
All Mass (g)	4.38	0.79	2.38 ± 0.83
Female Mass (g)	3.54	2.04	2.74 ± 0.40
Male Mass (g)	4.38	1.76	2.86 ± 0.62
Juvenile Mass (g)	2.44	0.79	1.44 ± 0.31

### 3.2 Parameters Effective on Body Temperature

**FIGURE 4:**
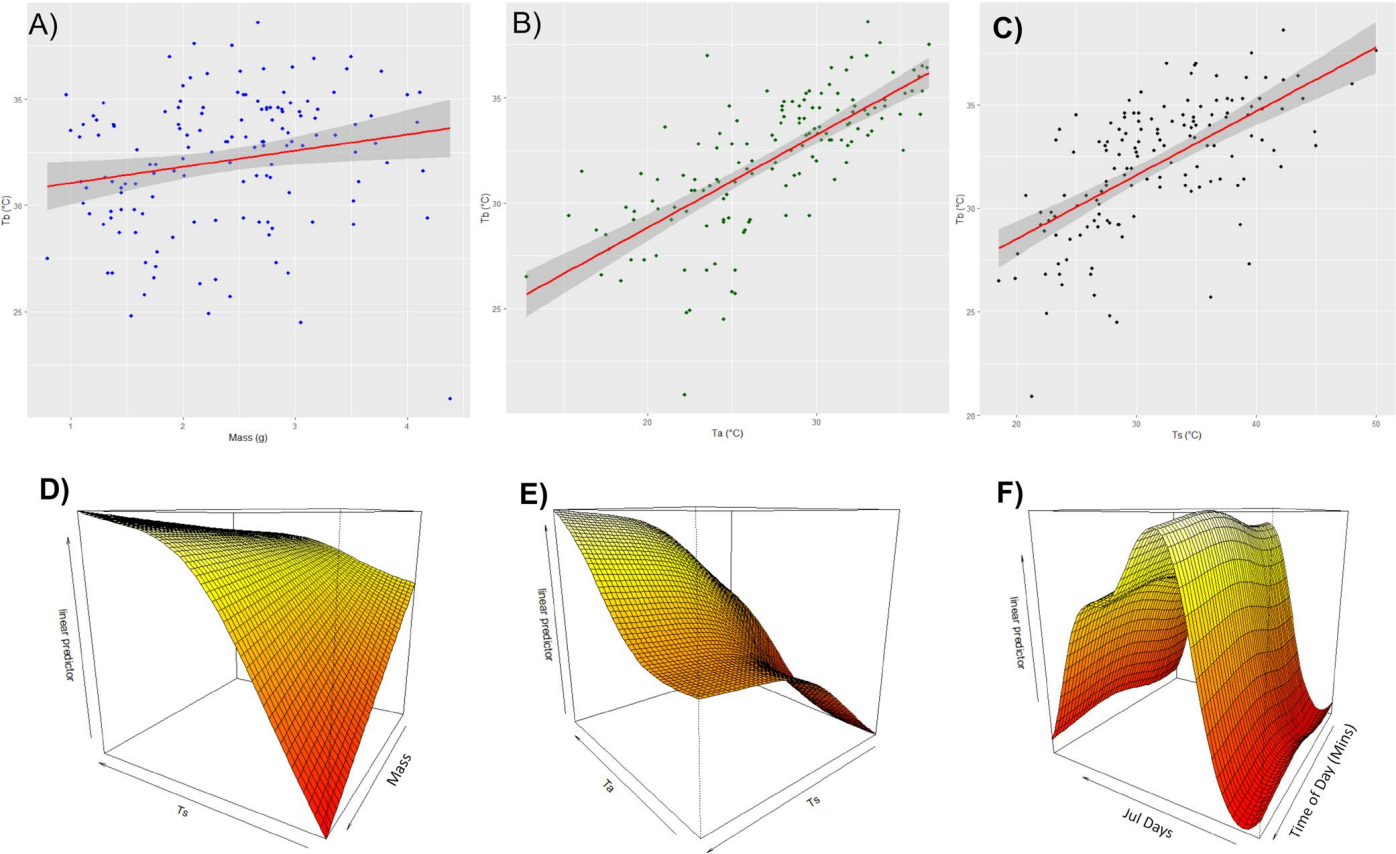
Top row shows the linear relations (A) between *T*_b_ and *Mass* (B) between *T*_b_ and *T*_a_ (C) between *T*_b_ and *T*_s_. The bottom row shows the 3D plots of two factors (D) *T*_s_ and *Mass* (E) *T*_a_ and *T*_s_ (F) Time of year (Julian days) and Time of day (in minutes).

According to the AIC results the first model had an AIC of 603.3 which decreased to 590.6 in the final selected model. The results of the final GAM model selected after elimination of nonimportant parameters with stepwise selection are shown in Table 3. Sex, substrate type, wind speed were not retained in the final model. Environmental parameters *T*_a_ and *T*_s_ were significant on their own (*p* < 0.05) while body mass was the sole important morphological parameter (*p* < 0.05). Although *Sol* was not statistically significant (*p* > 0.05) it was retained in the final model as one of the important parameters. Concerning interactions, the interaction between *T*_s_ and Mass was the only interaction included in the final model significant (*p* < 0.05). Julian day was the most significant temporal parameter in the model (*p* < 0.05) while time of day (in minutes) was not significant (*p* > 0.05). Plots of linear relations and 3D interactions can be seen in Figure 4.

**TABLE 3:** GAM results for factors effective on body temperature.

	edf	ref. df	*F*	*p*
*T*_a_	2.42	11	2.34	< 0.001***
*T*_s_	0.58	11	0.13	< 0.001***
*Sol*	0.47	11	0.083	0.13
*SVL*	1 × 10^−4^	11	~ 0	0.41
*Mass*	5.7 × 10^−4^	11	~ 0	0.007**
te (*Mass*, *T*_s_)	3.42	24	0.69	< 0.001***
Days (Time of year)	4.80	8	4.93	< 0.001***
Mins (Time of day)	1.00	8	0.24	0.10

Abbreviations: edf, estimated degrees of freedom; ref.df, estimated residual degrees of freedom.

** 0.001 < *p* < 0.01.

*** *p* < 0.001.

### 3.3 Thermal Preference and Effectiveness of Thermoregulation

**FIGURE 5:**
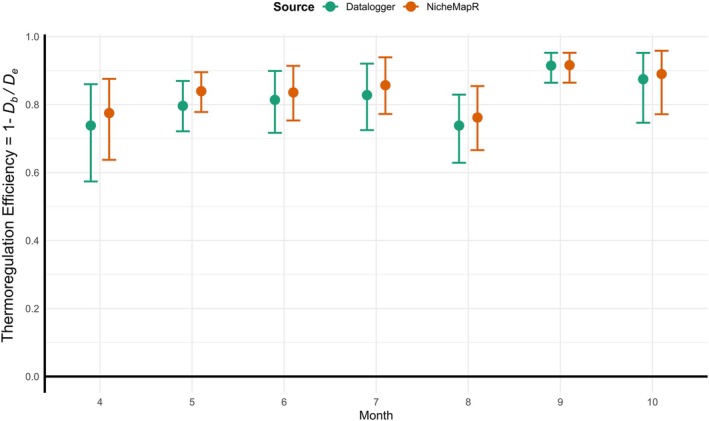
Thermoregulation efficiency values calculated for all months for NicheMapR and Datalogger derived parameters. The circles show medians with the upper and lower 95% confidence limits.

The results for thermal preference essays are also shown in Table 1. Kruskal–Wallis test results showed that there was not any significant difference between males, females, and juveniles for temperature preference (Kruskal–Wallis chi‐squared = 1.63, df = 2, *p* = 0.44). So the pooled distribution of all groups was used for *T*_pref_. The mean, maximum, and minimum temperatures in the temperature gradient were 32.10°C ± 3.2°C, 35.84°C and 26.48°C respectively. The calculated temperature preference range is compared with body temperature, microclimate temperatures recorded by dataloggers, and the NicheMapR microclimate model, as shown in Figure 3. Thermoregulation efficiency calculated from 1000 bootstraps were 0.77 ± 0.0095 for NicheMapR‐derived values and ~0.78 ± 0.009 for field datalogger values. Thermoregulation efficiency values by month are shown in Table 4. A *t*‐test showed a significant difference (*t* = 33.54, *p* < 0.001) however the detected difference was very small (0.013–0.014, 95% CI). The Nested ANOVA results showed that there is significant difference for both NicheMapR vs. Datalogger (*F*_1_ = 966.4, *p* < 0.001) and months (*F*_12_ = 1695, *p* < 0.001). On the other hand small differences are expected to be statistically significant for very large samples so it is also important to consult the results of the equivalence test. Although the NicheMapR showed higher thermoregulation efficiency than Datalogger derived values, efficiency values did not differ more than 5% from each other (TOST, *p* < 0.05) for all months (Figure 5).

**TABLE 4:** Thermoregulatory efficiency values calculated from Datalogger derived and NicheMapR modeled parameters for all months.

Month	Datalogger median eff. (95% CI)	NicheMapR median eff. (95% CI)
4	0.74 (0.57–0.86)	0.78 (0.64–0.88)
5	0.80 (0.72–0.87)	0.84 (0.78–0.90)
6	0.81 (0.72–0.90)	0.84 (0.75–0.91)
7	0.83 (0.72–0.92)	0.86 (0.77–0.94)
8	0.74 (0.63–0.83)	0.76 (0.67–0.85)
9	0.91 (0.86–0.95)	0.92 (0.86–0.95)
10	0.87 (0.75–0.95)	0.89 (0.77–0.96)

## 4 Discussion

### 4.1 Hypothesis 1: Abiotic Parameters Effective on Body Temperature

The results support our hypothesis, as for *Ophisops elegans* the significant abiotic parameters effective on body temperature in the field were air temperature and substrate temperature. Solar radiation was not significant according to the final model, hence for a small heliothermic lizard that depends on basking for thermoregulation this effect of solar radiation is an unexpected outcome as many studies showed the significant effect of solar radiation (Angilletta 2009; Barrett and O'Donnell 2023; Matthews et al. 2016; Porter and Gates 1969). It was also previously shown that the degree of artificial radiation was also an important factor in the effectiveness of thermoregulation in lizards (Aguado and Braña 2014). A previous study on another small lacertid species, *Parvilacerta parva* in a close region to the study area showed that the interaction between solar radiation and body length was a significant factor on body temperature (Şahin and Kuyucu 2021). This weak relation might be related to different factors: (1) being a heliothermic species *O. elegans* is mostly active on clear sunny days and there is a cluttering of samples at the higher end of the solar radiation distribution which might have resulted in a weaker linear relation. (2) Being a very small lacertid species, *O. elegans* is closer to the ground where the high intensity of substrate temperature (which is also strongly related to solar radiation) might be masking the direct effect of solar radiation. For small ectotherms the rapid cooling and heating due to low thermal inertia and more effective heat transfer from surface to body and through limbs closely follows the changes in microclimate (Dzialowski and O'Connor 1999; Herczeg et al. 2007). Gontijo et al. (2018) found that in a small mountain lizard, *Eurolophosaurus nanuzae*, body temperature did not show a direct relation with solar radiation. Other factors, such as the UV component can also influence the basking and shuttling behavior of lizards resulting in more complicated relationships (Conley and Lattanzio 2022). Although most diurnal lizard species are generally defined as heliotherms beforehand, our results point that smaller species like *O. elegans* might be inclined to thigmothermy due to the closer distance to ground. Additionally, despite the lack of a significant relation in the model, it does not mean that a diurnal species like *O. elegans* does not depend on solar radiation for thermoregulation as the ultimate source of heat gained from substrate and air depends is also solar radiation.

Although wind speed was counted among factors playing a significant role in thermoregulation (Ortega et al. 2017; Spears et al. 2024) it was not included among the important parameters in the final model. Previously Ortega et al. (2017) found that the body temperature of the cold specialist *Iberolacerta aurelioi*, a small species (approximately 5 cm) like *O. elegans* in the Lacertidae family, was affected by wind speed. Also, Pafilis et al. (2024) showed that wind speed and sheltering had a major impact on *Podarcis milensis*, another lacertid with similar size. On the other hand, these two species inhabit mountain ridges and islands, respectively, areas which are exposed to the effect of strong winds. In addition, experimental and field study results differ on the effect of wind on thermoregulation in various lizard species; for instance, in *Podarcis muralis* , in vitro temperature preference experiments showed that wall lizards selected higher temperatures in controlled conditions whereas wind speed did not have a significant effect on body temperature in the field (Spears et al. 2024), contrary to this, Virens and Cree (2022) showed that McCann's skink (*Oligosoma maccanni*) selected lower temperatures under wind treatment in experimental conditions. When we compare the results of these studies with the outcomes of this study, it seems that *O. elegans* might be responding differently to wind parameters compared to these other lizard species. This may be caused by the morphological characteristics of the organisms as well as their use of different thermoregulation strategies following these morphological characteristics and temperature preferences.

### 4.2 Hypothesis 2: Morphological Parameters Effective on Body Temperature

As support to our second hypothesis, mass was a significant factor by itself, additionally the interaction between mass and substrate temperature was also significant, pointing that for a small lacertid like *Ophisops elegans* body mass is indeed a very important factor for thermoregulation by conduction. This clear link between body mass and substrate temperature for thermal biology confirms our assumption that body size is indeed an important component of thermoregulation. Due to the lower thermal inertia, smaller lizard species heat and cool more rapidly compared to larger lizards, however lizards with large body size risk overheating if they stay too long in hot environments as they can not cool as rapid as small lizards when they retreat to shade (Ortega and Martín‐Vallejo 2019; Rubalcaba et al. 2019) additionally smaller sized lizards might also be more effective thermoregulators than larger lizards as shown on island populations by (Sagonas, Meiri, et al. 2013). On the other hand, small lizards also heat more quickly and their maximal body temperatures show more fluctuations (Seebacher and Shine 2004) and lizards which have smaller mass than 2–3 g cannot overcome the risk of overheating by shuttling in environments with rare sheltered cool microhabitats such as in the summer periods when temperature is at its annual maximum and thus should reduce or cease activity (Herczeg et al. 2007). Although overheating seems a bigger problem for larger ectotherms from a Newtonian perspective, the Geigerian perspective argues that larger ectotherms might be experiencing cooler environments due to the increasing effect of wind speed and decreasing effect of hot substrate and vice versa smaller ectotherms are expected to be involved in a warmer environment due to the higher effect of substrate temperature (Kearney et al. 2020).

In this study, the interaction between mass and substrate temperature, and thus conduction from the substrate was a very important factor in thermoregulation for *O. elegans* . On the surface it seems that the relation between substrate temperature and body temperature is linear (Figure 3b), and body temperature increases with substrate temperature. However, the results of the additive model show that when the relation between body size and substrate temperature is considered, this relationship becomes more complicated. From the 3D plot of interactions, it can be seen that small size has a great positive effect at low temperatures while the minimum point is where size is biggest at low temperatures (Figure 3e). In the area of thermal biology, there still exists a significant discrepancy between mathematical models, laboratory experiments, and field measurements as there are numerous factors that complicate the body temperature in the field such as behavior, other abiotic factors like humidity and stochastic climate patterns, and the heterogeneous thermal habitat structure (Gunderson and Leal 2016; Le Galliard et al. 2021; Neel and McBrayer 2018). For example, in the field ectotherms typically behaviorally adjust their activity and exploit microclimatic variability in their environment, which may be unnoticed in controlled laboratory experiments and despite its importance might be disregarded in correlational models (Fey et al. 2019; Goller et al. 2014; Gvoždík 2012). This diverse array of behavioral strategies may cause mismatches between observed and expected body temperatures in the field (Gunderson and Leal 2016; Neel and McBrayer 2018). On the other hand, the significant advances in mechanistic models that implement behavior and more precise physiological and morphological parameters and the usage of more advanced statistical models in the field close this gap step by step (Fei et al. 2012; Kearney and Porter 2020; Vickers and Schwarzkopf 2016). Additionally, humidity and water can significantly affect thermoregulation behavior as lizards in hot arid environments might tend to select lower temperatures and thermoregulate less efficiently when dehydrated (Le Galliard et al. 2021; Sannolo and Carretero 2019).

Sex was not an effective parameter on body temperature. Similarly, according to the previous study on other lacertids in Central Anatolia, there was not any relation between thermal biology and sex for relatively larger related species *Lacerta diplochondrodes* and *Parvilacerta parva* (Şahin and Kuyucu 2021). Several previous studies also found little effect of sex on thermoregulation in lacertid lizards (Aguado and Braña 2014; Sagonas, Meiri, et al. 2013).

### 4.3 Hypothesis 3: Thermoregulation Efficiency

This study confirms that *O. elegans* is a highly efficient thermoregulator; efficiency indices (*E*) obtained from field data and NicheMapR both exceed the 0.7 threshold. The results confirmed our assumption of efficient thermoregulation in *O. elegans* with *E* ≌ 0.77. This result is also consistent with the organism's roaming and burrowing in response to gradient temperatures in temperature preference studies, indicating that *O. elegans* regulates its body temperature with a shuttling strategy and this species is an effective thermoregulator according to the operative environmental temperatures by both NichemapR modeled values and datalogger records. In a meta‐analysis of 45 lacertid species, Ortega and Martín‐Vallejo (2019) reported that the range of thermoregulation efficiency changed between 0.43 and 0.95 with a 0.78 mean. Sagonas, Valakos, and Pafilis (2013) found that the thermoregulatory efficiency values were 0.64 and 0.72 for island and mainland populations of *Lacerta trilineata* in the Aegean, a medium‐sized lacertid lizard. In another study on the thermal biology of island and mainland populations of a small‐sized lizard, *Podarcis gaigeae* Sagonas, Meiri, et al. (2013) reported the thermoregulatory efficiency values as 0.87 and 0.72 respectively, with island populations with smaller body sizes having higher thermoregulatory efficiencies. Again, depending on biophysical models with NichemapR‐derived parameters for the Northern hemisphere Rubalcaba et al. (2019) mapped the predicted effectiveness of thermoregulation as near 0.7–0.8 range for dry skinned small (≤ 5 g) ectotherms in this region (central western Anatolia). However, body size is not the sole effective factor in the effectiveness of thermoregulation as (Sagonas et al. 2017) showed that microhabitat quality was more important on thermoregulation in mainland populations of 3 *Podarcis* species, on the other hand, all 3 populations conformed to the range predicted for their size with thermoregulation efficiency values ranging between 0.76 and 0.88. One potential limitation in our study is measuring the temperature from the dorsal region instead of taking cloacal measurements, this choice was due to the constraints of the setup (closed thermal gradient) and to reduce disturbance in thermal behavior of lizards. Although dorsal and cloacal show varying degrees of difference, they are highly correlated (Barroso et al. 2020) additionally we worked to reduce this difference further by taking the measurements with the same thermocouple used in field and the usage of a closed thermal gradient that does not use infrared heating.

Concerning the seasonality of thermoregulation, we also observed that thermoregulation efficiency showed significant changes between seasons, with the highest efficiency attained in September and October. This is an expected result as while temperature deviation from optimal set points is very high during these periods, lizards were able to keep their body temperature close to the set point range (Figure 3c,d). In warmer seasons, environmental temperatures are close to set point ranges, but in colder seasons, efficiency is maintained through behavioral adjustments and physiological acclimatization (McMaster and Downs 2013). A similar pattern was also reported for a sceloporine mesquite lizard, *Sceloporis grammicus* which also showed highest thermoregulation efficiency in autumn, and also for skink, *scincella lateralis* which showed highest efficiency in autumn/winter (Rivera‐Rea et al. 2023; Parker 2014).

### 4.4 Hypothesis 4: Accuracy of NicheMapR Modeled Parameters

Microclimate modeling tools such as Microclima and NicheMapR have been used extensively and successfully in several studies including investigating heat and water budgets of ectotherms (Kearney et al. 2020), inferring the response of phenology, morphology, behavior, and physiological tolerances to present and future climatic constraints (Huang et al. 2020; Mader et al. 2022; Turner and Maclean 2022) and implementation of physiological parameters to species distribution models (Toro‐Cardona et al. 2023). Although NichemapR and datalogger recorded values were close to each other for some months we also observed significant differences in certain periods (Figure 3a). One reason might be NicheMapR's inability to detect extremes in certain seasons. The higher discrepancy that we have observed in May might be due to the fact that this period is the wettest in Central Anatolia, when high humidity and rapid growth of plants highly affect the microclimate temperature, an effect that can be overlooked by modeling software like NicheMapR. Thus there is a need for additional studies to validate the accuracy of modeling software like NicheMapR and Microclima. On the other hand, we also observed that modeled and field values for efficiency were very close despite the difference in temperature measures. Thermoregulation efficiency prioritizes optimal intervals rather than point values; if observed temperatures fall inside set points it means 0 deviation values, which results in a smaller difference between efficiencies if temperature difference is not very high. This agreement between field and modeled data supports the validity of mechanistic modeling. Although seasonal deviations occurred, the negligible biological difference between the calculated indexes demonstrates that NicheMapR is a robust tool for assessing the thermal ecology of small lizards.

## 5 Conclusion

In conclusion, this research provides a detailed characterization of the thermal niche of a central population of *O. elegans* . Our findings showed that substrate and air temperatures were the primary drivers of body temperature despite the lack of a significant direct effect of solar radiation (H1); it was also confirmed that body mass significantly modulates thermal dynamics through the effect of conduction with ground (H2). This local population of *O. elegans* also maintains a rather high thermoregulatory efficiency (H3). Finally, our results validate NicheMapR as a reliable tool for predicting thermal biology indices despite some small seasonal discrepancies caused by the complexity of environmental processes (H4).

The distribution of *O. elegans* covers a wide array of ecological regions from Mediterranean coastal areas with relatively stable seasons to continental regions with larger seasonal and daily fluctuations. As the population that we have studied is from one of the continental central regions, this efficiency value may differ in other regions depending on environmental conditions and intraspecific variation in behavior. Future research exploring the relationship between physiological and ecological factors across various populations and habitats will enhance our understanding of how lizards like *O. elegans* adjust to shifting environmental conditions, providing crucial insights for their conservation in the face of climate change.

## Author Contributions

**Sezer Dıblan:** conceptualization (equal), data curation (equal), formal analysis (equal), investigation (equal), methodology (supporting), visualization (supporting), writing – original draft (equal), writing – review and editing (equal). **Arda Cem Kuyucu:** conceptualization (equal), data curation (equal), formal analysis (equal), investigation (equal), methodology (lead), visualization (lead), writing – original draft (equal), writing – review and editing (equal). **Mehmet Kürşat Şahin:** conceptualization (supporting), investigation (supporting), methodology (supporting), writing – review and editing (supporting). **Selim Sualp Çağlar:** supervision (equal).

## Funding

The authors have nothing to report.

## Conflicts of Interest

The authors declare no conflicts of interest.

## Supporting information


**Data S1:** ece373518‐sup‐0001‐Supinfo.rar.

## Data Availability

The data is available in Supporting Information.
